# The Restaurant Food Hot Potato: Stop Passing it on—A Commentary on Mah and Timming’s, ‘Equity in Public Health Ethics: The Case of Menu Labelling Policy at the Local Level’

**DOI:** 10.1093/phe/phu046

**Published:** 2015-01-19

**Authors:** Kathryn L. MacKay

**Affiliations:** University of Birmingham

## Abstract

In the case discussion, ‘Equity in Public Health Ethics: The Case of Menu Labelling Policy at the Local Level’ (2014), Mah and Timming state that menu labelling would ‘place requirements for information disclosure on private sector food businesses, which, as a policy instrument, is arguably less intrusive than related activities such as requiring changes to the food content’. In this commentary on Mah and Timming’s case study, I focus on discussing how menu-labelling policy permits governments to avoid addressing the heart of the problem, which is high-calorie, high-sodium restaurant food. Menu labelling policy does not address food content in a way that is meaningful for change, instead relying on individuals to change their behaviour given new information. Besides having questionable efficacy, this raises concerns about moralizing food choices.

In the case discussion, ‘Equity in Public Health Ethics: The Case of Menu Labelling Policy at the Local Level’ (2015), Mah and Timming state that menu labelling would ‘place requirements for information disclosure on private sector food businesses, which, as a policy instrument, is arguably less intrusive than related activities such as requiring changes to the food content’. In this commentary on Mah and Timming’s case study I will focus on discussing how menu-labelling policy permits governments to avoid addressing the heart of the problem, does not address food content in a way that is meaningful for change and raises concerns about moralizing food choices.

## Menu Labelling—Trivia or Tool

Menu labelling provides an example of the tension between the demands of consumers to have information, and the goals of industry to find profits. Menu labelling enjoys a high rate of support from consumers (Ontario Ministry of Health and Long-Term Care, 2013; Mah and Timmings, 2015). Many people want to know what is in the food that they are eating or feeding their children, and there is a strong argument for the view that they should be able to easily access this information when they want it. Menu labelling is also seen by the government as a tool to change the choices of consumers that the government does not like. One reason that the government does not like some food choices at restaurants is the link between eating out frequently and becoming overweight or obese, and these bodily states are a concern for government because of the costs to various health and social services caused by overweight or obese citizens, economic costs from lost productivity from same, and so on. However, industry may not welcome menu labelling because disclosure of information about food content can be seen as a threat to profits (via lost revenue from consumers and the initial costs of changing menu boards, etc).

Mah and Timming suggest that the motivation behind menu labelling is to provide facts: to disclose the truth about food content to consumers. Transparency of this kind is certainly important, but simple disclosure is not the end goal of menu labelling efforts. Menu labelling is not introduced to provide interesting trivia to consumers; the passage of menu-labelling legislation is an instrumental goal. Such legislation is intended to educate people, empower them or aid them to make different food choices, with a focus on preserving customer autonomy (Tengland, 2012). Guiding individuals away from higher-calorie options toward lower-calorie options through the provision of information, and therefore causing people to make different food choices, is the ultimate aim of menu labelling efforts. This is implied, if not stated outright, in justifications for menu labelling legislation, debates on the topic, and in the myriad studies on its effectiveness (Ebel *et al.*, 2011; Ontario Ministry of Health and Long-Term Care, 2013; Mah and Timmings, 2015).

The policy aim is to achieve the outcome that people eating at restaurants will consume fewer calories, but rather than addressing the source of the calories in the food (i.e. charging the industry with making changes to food content), menu labelling puts the responsibility for achieving this goal on the shoulders of consumers. As a behaviour-change measure, menu labelling is a form of soft paternalism, aiming to alter people’s food-ordering choices by changing the status of some foods to make them undesirable, instead of making the food healthier or taking it off the menu outright (Rabin, 2008). The justification, as Mah and Timmings state, is that putting the responsibility on consumers is less intrusive than putting it on industry, though researchers have found that it is also less effective (Ebel *et al.*, 2011).

## Right and Wrong Orders—Moralizing Food

Mah and Timmings argue that menu labelling preserves consumer autonomy by simply providing information, and is therefore less coercive than changing food recipes. However, a foreseeable consequence of menu labelling is that it would result in the attachment of moral status to food orders. Similar to the stop-light system of nutrition labelling on packaged food, restaurant orders with fewer calories and less sodium would be ‘good’—in a sense that combines moral, social and health meanings of ‘goodness’—while those higher in both calories and sodium would be bad—morally, socially and health-wise (for an example of this see reference Change 4 Life, 2014).

Imagine that menu labelling had been established and chain restaurants were required to post calorie and sodium amounts beside menu items. If people of size were to go into one of these restaurants and order one of their favourite things, which also happened to be one of the top-five high-calorie and -sodium menu items, they would likely face judgment by the staff and other customers for their choice. They would also be more likely to be blamed for being large in body based on that food order, regardless of their actual health status (Puhl and Heuer, 2010). It is conceivable that no one with a larger body would order any of those top-five menu items. Further, a person slight in body who ordered the same thing would likely face less judgment, as their socially appropriate weight would provide moral permission to eat such food. Those people who did order one of the five, regardless of their body size, would perhaps see what they were doing as something wrong or deviant.

I do not think that public health agencies aim to encourage the creation of judgmental eating environments (especially with the spectre of disordered eating lurking in the shadows), but this is a reasonably foreseeable result of menu labelling legislation. Menus would quickly be parsed into the socially and morally right things and wrong things to order based on caloric and sodium content. Perhaps this is one of the intentions of such a policy; by creating a category of ‘wrong things to order’, consumer demand may be able to influence restaurants to provide different menu options in the ‘right things’ category, or reformulate old favourites that have fallen into the ‘wrong things’ category. If changes to food content are a hoped-for outcome of menu labelling legislation, then it is another way in which governments are putting their own work on the shoulders of consumers.

## With a Side of Nanny State

The argument about the degree of paternalism involved in government intervention hangs upon whether people desire all of the calories and sodium that are currently served in restaurant food. If we assume that when people go out to eat, they go with the desire to eat all the calories and sodium that are currently in some dishes at some restaurants, then offering them menu labels or interfering with the food recipes to encourage them to eat fewer calories and less sodium could be considered coercive. Arguments of this kind have been presented against proposed restaurant-focussed public health interventions either for forcing unwanted information upon consumers or making them eat food that is healthier than they desired (Von Tigerstrom, 2010).

However, I have a strong intuition that the active desire of a diner at a restaurant is not to eat as many calories or milligrams (mg) of sodium as are in some of their favourite meals, but rather to eat tasty food. If a meal had the potential to be lower in calories and sodium and still be tasty, as many restaurant meals already are, then the consumers would have their desires for the particular food satisfied without requiring all of the calories or sodium.

A report by the Centre for Science in the Public Interest compiled calorie and sodium information from 28 of Canada’s 100 most popular chain restaurants, some of which also have locations in the USA or worldwide (Jeffery and Cappello, 2012). While reading this report, one wonders whether when people order the Italian-style nachos appetizer at Pizza Hut they desire to eat 2,320 calories, or actually just desire some nachos before the pizza arrives? Do people desire to eat 2,200 calories when they order the full back ribs at East Side Mario’s, or do they just feel like eating BBQ tonight? The amounts of sodium in these dishes are extremely high. There are some who argue that people may try to maximize their calories-per-dollar when purchasing food on a limited budget. The same argument has not been offered for sodium, presumably because that would be ridiculous. To illustrate, Pizza Hut’s Italian-style nachos have 2,010 mg of sodium, and the full back ribs at East Side Mario’s contain 4,040 mg (which is 387% of the daily recommended amount of 1,500 mg for an adult) (Jeffery and Cappello, 2012).

It is difficult to know what people desire when they make certain decisions, and whether a person really does desire 2,320 calories and 2,010 mg of sodium as an appetizer or in fact simply desires some nachos. However, intuition suggests that if one were offered a plate of 2,320 calories with 2,010 mg of sodium in the form of grey wafers, one’s mouth would not water. If one were offered a plate of ‘Italian-style’ nachos with lower amounts of calories and sodium, one’s appetite would likely still be whetted. It is not the calories and sodium that one desires; it is the tasty food.

Some, particularly the food industry, may object that without the calories or sodium the food would not be tasty, and therefore the calories and sodium are required for the food to be desired, or it is in fact the calories and sodium that one desires because they are the essence of the tasty food. However, this position is a weak argument for the status quo. A moment’s reflection reveals that nachos can be delicious without 2,320 calories and 2,010 mg of sodium because experience has provided, at one time or another, nachos created by a person at home or at a restaurant that were delicious and had neither of these amounts of calories and sodium. If the restaurant industry is unable to make its food delicious without these components, then there may be important problems with the quality or production process of the food it serves to people.

## Conclusions

Noticing the distinction between a person’s desire to eat thousands of calories and a person’s desire to eat some nachos provides room for policy interventions that hold the restaurant industry accountable for the food they serve. Policies like those which call for menu reformulation respect the consumer’s autonomy by preserving the range of restaurant menu options and avoiding making some items morally or socially unavailable, while having a better chance of achieving the desired behaviour change (a reduction in calorie and sodium intake). However, by passing the responsibility for changing the food that people eat from the food industry to the consumers with menu labelling, government ducks the problem of consumers eating too many calories and too much sodium. Policy interventions that shift responsibility for poor food choices firmly to the consumer not only hold the potential to fail to create change, but risk exacerbating various issues around eating, with important implications for physical and mental health (Puhl and Heuer, 2010).

Though the goal that people should change their ordering behaviour on an individual basis to consume fewer calories has been discussed in debates about menu labelling, the moralizing of food has not garnered much attention. Menu labelling may encourage negative judgment of people’s eating habits and increase the experience of stigma for many, turning eating environments into hostile spaces, all the while avoiding addressing the actual source of the problem: high-calorie and high-sodium restaurant food. Addressing the issue head-on by requiring restaurants to lower calorie and sodium amounts to a certain threshold across the board may be a way to preserve the entire menu’s worth of choices for consumers, without creating ‘good’ and ‘bad’ food orders.
